# Performance of Immunoglobulin G Serology on Finger Prick Capillary Dried Blood Spot Samples to Detect a SARS-CoV-2 Antibody Response

**DOI:** 10.1128/spectrum.01405-21

**Published:** 2022-03-10

**Authors:** Aidan M. Nikiforuk, Brynn McMillan, Sofia R. Bartlett, Ana Citlali Márquez, Tamara Pidduck, Jesse Kustra, David M. Goldfarb, Vilte Barakauskas, Graham Sinclair, David M. Patrick, Manish Sadarangani, Gina S. Ogilvie, Mel Krajden, Muhammad Morshed, Inna Sekirov, Agatha N. Jassem

**Affiliations:** a British Columbia Centre for Disease Control, Vancouver, British Columbia, Canada; b School of Population and Public Health, University of British Columbia, Vancouver, British Columbia, Canada; c Department of Pathology and Laboratory Medicine, University of British Columbia, Vancouver, British Columbia, Canada; d Department of Experimental Medicine, University of British Columbia, Vancouver, British Columbia, Canada; e Department of Pathology and Laboratory Medicine, British Columbia Children’s and Women’s Hospital, Vancouver, British Columbia, Canada; f Vaccine Evaluation Center, British Columbia Children’s Hospital Research Institute, Vancouver, British Columbia, Canada; g Women's Health Research Institute, Vancouver, British Columbia, Canada; h Department of Pediatrics, British Columbia Children’s Hospital, Vancouver, British Columbia, Canada; Texas A&M University

**Keywords:** SARS-CoV-2, dried blood spots, seropositivity, diagnostic accuracy, vaccine evaluation, public health, COVID-19, data analysis, epidemiology

## Abstract

We investigate the diagnostic accuracy and predictive value of finger prick capillary dried blood spot (DBS) samples tested by a quantitative multiplex anti-immunoglobulin G (IgG) assay to detect severe acute respiratory syndrome coronavirus 2 (SARS-CoV-2) antibodies after infection or vaccination. This cross-sectional study involved participants (*n* = 6,841) from several serological surveys conducted in nonhospitalized children and adults throughout 2020 and 2021 in British Columbia (BC), Canada. Analysis used paired DBS and serum samples from a subset of participants (*n* = 642) prior to vaccination to establish signal thresholds and calculate diagnostic accuracy by logistic regression. Discrimination of the logistic regression model was assessed by receiver operator curve (ROC) analysis in an *n* = 2,000 bootstrap of the paired sample (*n* = 642). The model was cross-validated in a subset of vaccinated persons (*n* = 90). Unpaired DBS samples (*n* = 6,723) were used to evaluate anti-IgG signal distributions. In comparison to paired serum, DBS samples from an unvaccinated population possessed a sensitivity of 79% (95% confidence interval [95% CI]: 58 to 91%) and specificity of 97% (95% CI: 95 to 98%). ROC analysis found that DBS samples accurately classify SARS-CoV-2 seroconversion at an 88% percent rate (area under the curve [AUC] = 88% [95% CI: 80 to 95%]). In coronavirus disease 2019 (COVID-19) vaccine dose one or two recipients, the sensitivity of DBS testing increased to 97% (95% CI: 83 to 99%) and 100% (95% CI: 88 to 100%). Modeling found that DBS testing possesses a high positive predictive value (98% [95% CI: 97 to 98%]) in a population with 75% seroprevalence. We demonstrate that DBS testing should be considered to reliably detect SARS-CoV-2 seropositivity from natural infection or vaccination.

**IMPORTANCE** Dried blood spot samples have comparable diagnostic accuracy to serum collected by venipuncture when tested by an electrochemiluminescent assay for antibodies and should be considered to reliably detect seropositivity following SARS-CoV-2 infection and/or vaccination.

## INTRODUCTION

The ongoing global vaccination campaign to immunize populations against severe acute respiratory syndrome coronavirus 2 (SARS-CoV-2) infection, which causes coronavirus disease 2019 (COVID-19), represents the largest primary prevention effort undertaken in public health since the Global Polio Eradication Initiative (GPEI) (1). Lessons learned from the GPEI highlight the value of measuring population-level vaccine-elicited immunogenicity, as the humoral response can differ between doses, age groups, vaccine formulations, and viral strains (1). Evaluation of immunogenicity against an antigen presented to the immune system from natural infection or vaccination requires serological testing from whole blood to quantify the concentration of specific antibodies (2). Whole blood may be collected in large volumes by venipuncture or alternatively in small volumes from capillary beds by needle prick. Colloquially referred to as “dried blood spots” (DBS), capillary blood collection utilizes sterile filter paper to collect and store ∼350 μL of dried blood in several nonoverlapping spots (3). Numerous population-level studies have adopted DBS sampling for surveillance of chronic and acute viral infections (e.g., hepatitis B or C) (4) due to its comparable diagnostic accuracy to samples collected by venipuncture, minimal invasiveness (suitability for needle-hesitant persons), reduced cost, and the ability to rapidly deploy in resource-limited settings (5).

The size and scale of the COVID-19 vaccination campaign raises logistical challenges in measuring SARS-CoV-2 vaccine-elicited antibody response at the population level. The requirement for high-volume serologic testing to detect vaccine-elicited immunogenicity represents a particular challenge, as whole-blood specimen collection by venipuncture does not easily scale up (3, 6). Whole blood collection in the form of serum or plasma requires a trained phlebotomist, specific collection tubes, and cold chain logistics. DBS sampling is a cost-effective and promising alternative, which can occur by self-collection, eliminating the need for trained personnel. Collection cards are stable at ambient temperatures for up to 2 weeks, simplifying transport, and can be stored in large quantities using less space (7). To optimize the benefits of DBS collection, the downstream clinical assay must have multiplex capacity across a wide dynamic range because SARS-CoV-2 has several antigenic targets (structural proteins), and a large-fold difference in antibody concentration occurs between naturally infected and vaccinated persons (8, 9). The SARS-CoV-2 genome encodes four major structural proteins: the spike (S) glycoprotein, which is responsible for cellular entry, the envelope (E) protein, which enables cellular fusion, the membrane (M) protein, which binds the other structural proteins, and the nucleocapsid (N) protein, which has a multifunctional role in transcription and virion assembly (10). The S and N proteins serve as the best antigenic targets for serological testing because S binds to the host receptor angiotensin converting enzyme 2 (ACE2) via the receptor-binding domain (RBD) and is the primary target of neutralizing antibodies (11, 12). The concentration of N protein exceeds that of other viral genes during infection due to the unique coronavirus replication mechanism of discontinuous negative-strand transcription (13). To understand the potential of DBS sampling to detect SARS-CoV-2 seropositivity at the population level we i) evaluated the diagnostic accuracy of DBS tested by Meso Scale Discovery’s quantitative multiplex anti-IgG electrochemiluminescence assay (DBS-MSD) compared to paired serum samples in study participants prevaccination (*n* = 642; 28 positive and 614 negative) from BC, Canada; ii) cross-validated the diagnostic accuracy in a random sample of participants before vaccination (*n* = 30) or 3 to 6 weeks after dose one (*n* = 30) or two (*n* = 30) of receiving a COVID-19 vaccine; and iii) modeled the predictive performance of DBS-MSD testing in a theoretical population (*n* = 10,000) with stratified COVID-19 seroprevalence of 15, 45, and 75%.

## RESULTS

### Anti-SARS-CoV-2 IgG linear range.

In the tested DBS samples (*n* = 6,841), no drop out was observed on the MSD assay, and all values were above the antigen-specific lower limit of detection (anti-S = 0.049, anti-N = 0.046, anti-RBD = 0.035 AU/mL) (14). Linear regression found a proportionate relationship between signal intensity and anti-IgG concentration (arbitrary units [AU]/mL) across a 3- to 4-log_10_ range or ∼3-fold change (Fig. S1 in the supplemental material) per antigen (S: *R*^2^ = 0.99, *P* < 0.001; N: *R*^2^ = 0.95, *P* < 0.001; RBD: *R*^2^ = 0.97, *P* < 0.001). Anti-RBD results were not interpreted for DBS samples, as they were found to be highly correlated with anti-S signals (Pearson’s correlation, *r* = 95%, 95% confidence interval [95% CI] of 92 to 97%), indicating collinearity (15).

### Threshold determination and diagnostic accuracy.

**FIG 1:**
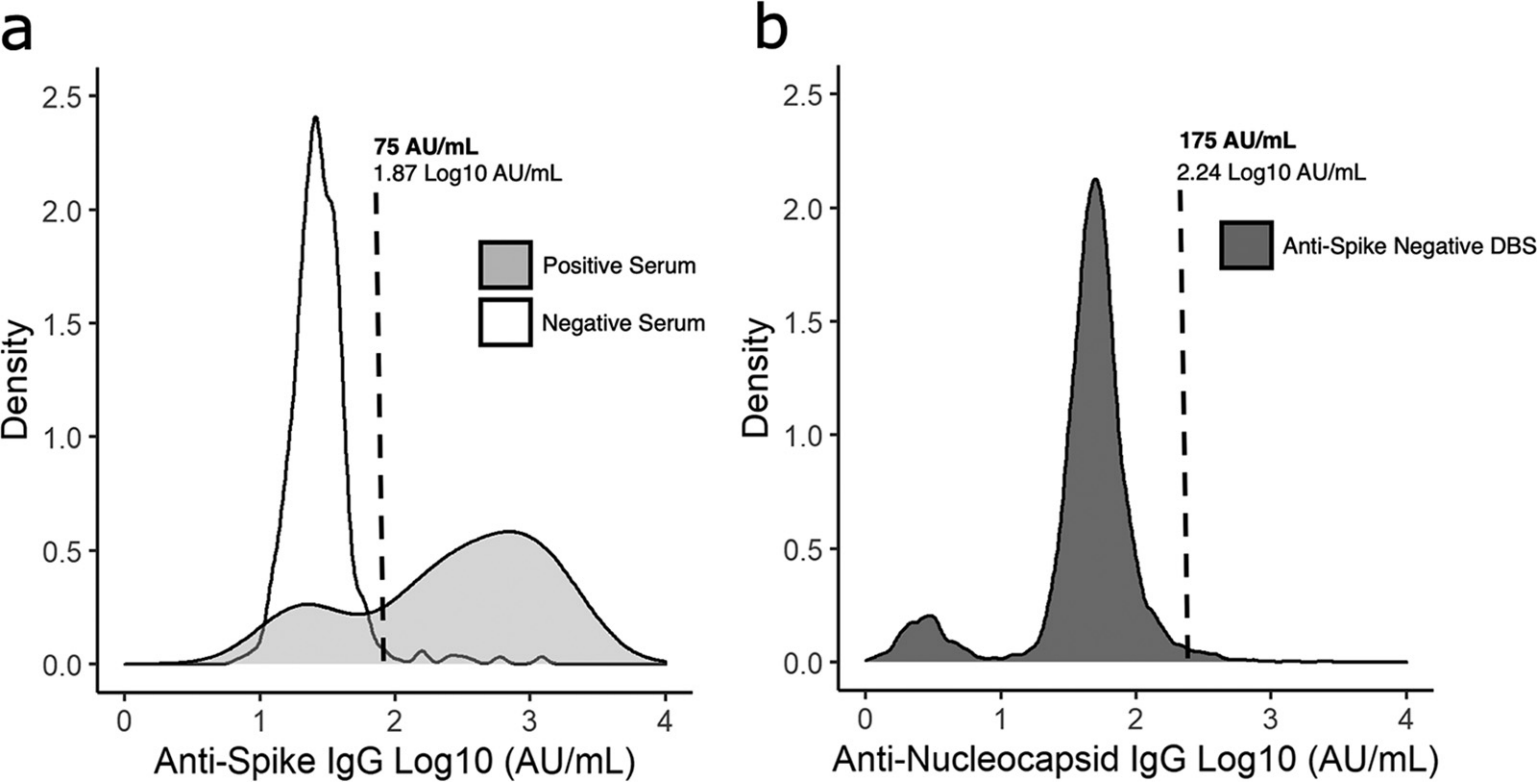
Signal distributions of SARS-CoV-2 anti-spike (S) and anti-nucleocapsid (N) IgG collected by DBS and serum and tested with an MSD assay. (a) Participant DBS and paired serum samples (*n* = 642) were tested by MSD assay for anti-S, anti-N, and anti-receptor-binding domain (RBD) IgG. DBS-MSD anti-S signals were stratified by MSD test results on paired serum samples (white: paired serum positive; gray: paired serum negative). A sample was classified as paired serum positive when greater than or equal to two of three target signals exceeded the manufacturer-recommended thresholds (S = 1,960, N = 5,000, and RBD= 538). A threshold of ≥75 AU/mL (95% CI: 55 to 95 AU/mL) was set for anti-S DBS samples tested on MSD, as it discriminates paired serum positives from negatives. In a random sample of serum positives, 77% of paired DBS-MSD samples are expected to have values greater than or equal to 75 AU/mL (one sample *t* test, *P* = 0.77, sensitivity = 77%). (b) All anti-N DBS-MSD samples tested at the British Columbia Centre for Disease Control (BCCDC) to 21 May 2021 were restricted to those with DBS-MSD anti-S of <75 AU/mL (*n* = 6,723; dark gray). A threshold of ≥175 AU/mL (95% CI: 162 to 188 AU/mL) was set for anti-N DBS samples tested on MSD, as the probability of classifying an anti-S negative DBS-MSD sample anti-N positive equals 5% (one sample *t* test, *P* = 0.05, specificity= 95%). DBS-MSD samples were classified positive if anti-S signal was ≥75 AU/mL and anti-N signal was ≥175 AU/mL or anti-S signal was ≥75 AU/mL and anti-N signal was <175 AU/mL. Samples with anti-S signal <75 AU/mL and anti-N signal ≥175 AU/mL were classified as negative.

**FIG 2:**
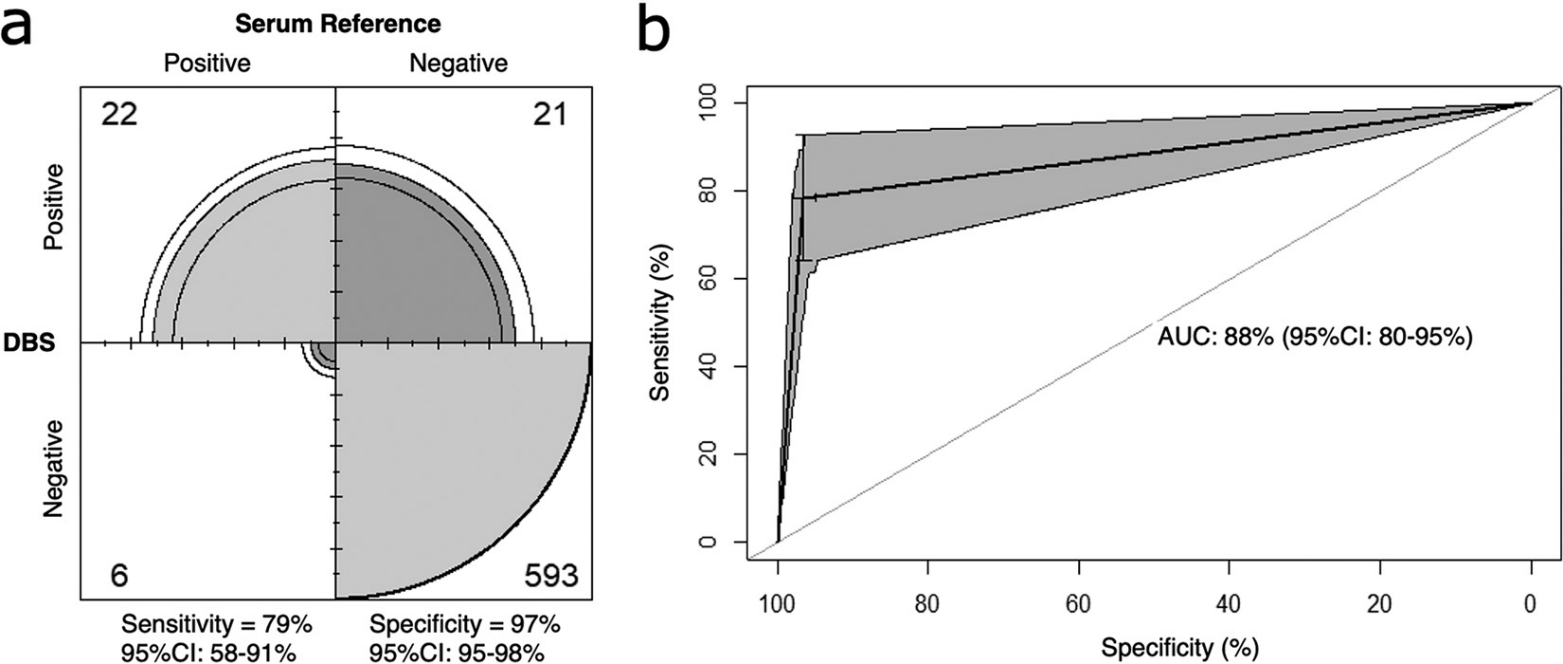
Confusion matrix and receiver operating characteristic curve analysis of DBS-MSD test result in comparison to the paired serum reference. (a) Frequency of DBS-MSD results are reported in comparison to the reference and used to calculate diagnostic accuracy (sensitivity and specificity) by logistic regression. In comparison to the paired serum reference, DBS-MSD possesses a sensitivity of 79% (95% CI: 58 to 91%) and specificity of 97% (95% CI: 95 to 98%); the gray area shows the proportion of participants by cell, and black lines represent the 95% confidence interval. No evidence of similarity between the marginal outcome probability was observed (McNemar test, *P* < 0.007). (b) Receiver operator characteristic curve analysis in an *n* = 2,000 bootstrap sample was used to quantify the discrimination (predictive ability) of a DBS-MSD test in comparison to the reference. DBS-MSD was found to accurately discriminate natural SARS-CoV-2 seroconversion at an 88% (95% CI: 80 to 95%) rate.

Signal target thresholds for DBS-MSD results were established at 75 AU/mL (95% CI: 55 to 95 AU/mL) for anti-S (Fig. 1a) and 175 AU/mL (95% CI: 162 to 188 AU/mL) for anti-N IgG (Fig. 1b), based on the observed signal distributions. The anti-S and anti-N thresholds were set to maximize sensitivity and specificity. The anti-S distribution showed that 77% of SARS-CoV-2 serum-positive samples from naturally infected participants have a DBS signal greater than or equal to the threshold (one sample *t* test, *P* = 0.77, sensitivity = 77%). The anti-N distribution estimates that 95% of anti-S-negative DBS samples have a signal less than or equal to the threshold (one sample *t* test, *P* = 0.05, specificity = 95%). Applying these thresholds to the same data set (*n* = 642), DBS-MSD achieved a sensitivity of 79% (95% CI: 58 to 91%) and specificity of 97% (95% CI: 95 to 98%) in an unvaccinated population compared to the paired serum reference (Fig. 2a). A diagnostic threshold was not assigned for anti-RBD IgG, as the signal exhibits colinearity with anti-S and, therefore, does not offer supplementary diagnostic information.

### Discrimination.

ROC analysis yielded an area under the curve of 88% (95% CI: 80 to 95%) (Fig. 2b). DBS samples resulted on MSD will accurately classify SARS-CoV-2 seroconversion in an unvaccinated population at an 88% percent rate.

### Cross-validation.

**FIG 3:**
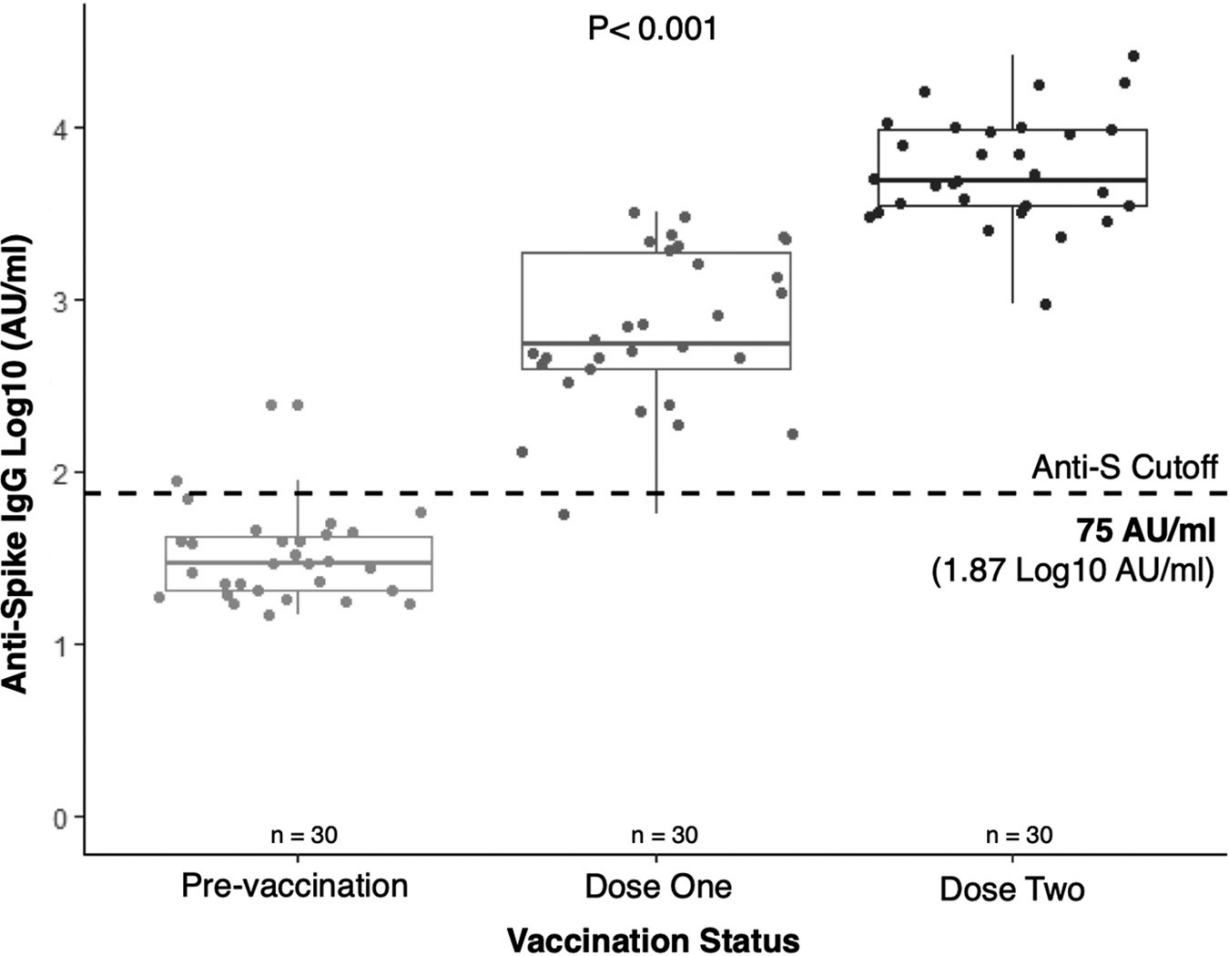
Boxplot stratified by participant COVID-19 vaccination status; *n* = 30 unpaired participants were randomly sampled per strata from the PREVENT-COVID study (Table 2), and their DBS sample was tested on the MSD assay. In the prevaccination strata, the anti-S cutoff of ≥75 AU/mL (1.87 log_10_ AU/mL) classified three participants as false positive for a specificity of 90% (95% CI: 73 to 98%). The sensitivity of the DBS-MSD test increased in the dose-one and dose-two groups compared to the estimate from unvaccinated (naturally infected) persons (Fig. 1). In participants with one dose of COVID-19 vaccine, 1 of 30 samples was classified as false negative for a sensitivity of 97% (95% CI: 83 to 99%). No false negatives were detected in participants who received two doses (sensitivity of 100% [95% CI: 88 to 100%]). DBS samples were collected from dose one or dose two recipients three to six weeks after administration of their vaccine. A two-way analysis of variance (ANOVA) found that a positive relationship exists between vaccine dose and anti-S IgG concentration; the true difference in mean antibody concentration does not equal zero (*P* < 0.001).

In unvaccinated participants from the PREVENT-COVID study, 3 of 30 samples were classified as false positive because their anti-S IgG concentration exceeded the cutoff of 75 AU/mL (1.87 log_10_ AU/mL). Therefore, 27 of 30 samples were classified correctly as negative for a specificity of 90% (95% CI: 73 to 98%) (Fig. 3). In persons with one dose of a COVID-19 vaccine, 1 of 30 samples was classified as false negative, indicating a sensitivity of 97% (95% CI: 83 to 99%) (Fig. 3). In two-dose recipients, no samples were classified as false negatives, for a sensitivity of 100% (95% CI: 88 to 100%) (Fig. 3). Samples from vaccinated participants were collected 3 to 6 weeks after administration of dose one or two. A positive relationship between vaccination and anti-S IgG was observed, which makes the DBS-MSD test more sensitive than when used to test for natural infection (Fig. 3). The 95% confidence intervals for sensitivity or specificity calculated in unvaccinated participants overlap with those of one- or two-dose recipients, indicating no significant difference in the test’s diagnostic accuracy (Fig. 2a and Table 1).

**TABLE 1:** Sensitivity and specificity estimates were averaged between unvaccinated (*n* = 642) and vaccinated participants (*n* = 90) and used to model the predictive value of the DBS-MSD test in a theoretical population of *n* = 10,000 persons with stratified seroprevalence of 15, 45, or 75%

Averaged estimate^*a*^		Expected seroprevalence^*a*^ (%)		
Sensitivity estimate (95% CI)	Specificity estimate (95% CI)	15% estimate (95% CI)	45% estimate (95% CI)	75% estimate (95% CI)
92% (76–97%)	94% (84–98%)	PPV: 73% (71–75%)	PPV: 93% (92–93%)	PPV: 98% (97–98%)
		NPV: 99% (99–100%)	NPV: 93% (93–94%)	NPV: 80% (78–81%)

a Point estimates and 95% CIs are reported. Individual estimates are available in Table S1 and Table S2 in the supplemental material.

### Prevalence and predictive value.

In a theoretical population of 10,000 persons with a seroprevalence of 75%, a positive test result predicts true seropositivity at a 98% rate (positive predictive value [PPV] = 98%; 95% CI: 97 to 98%) (Table 1). A negative test result predicts a seronegative response at an 80% rate (negative predictive value [NPV] = 80%; 95% CI: 78 to 81%). At lower seroprevalences of 15 and 45%, the PPV of DBS-MSD decreased, and the NPV increased (PPV_15_ = 73% [95% CI: 71 to 75%] and NPV_15_ = 99% [95% CI: 99 to 100%]; PPV_45_ = 93% [95% CI: 92 to 93%] and NPV_45_ = 93% [95% CI: 93 to 94%]) (Table 1).

## DISCUSSION

The diagnostic accuracy of MSD is comparable to that of the Roche Elecsys assay (16) and other commercial platforms (17) for surveillance of SARS-CoV-2 seroconversion in DBS-collected specimens. The MSD assay has the unique benefit of testing anti-S and anti-N IgG in a single multiplex reaction (18). Tests performed on DBS samples characteristically exhibit high specificity and low sensitivity, attributable to low analyte concentration, variance in sample collection practices, or time since antigen exposure (19). We show that despite the expected disadvantage of low analyte concentration, DBS-MSD testing possesses a strong PPV when implemented in a context with high SARS-CoV-2 seroprevalence (e.g., in an outbreak setting or to evaluate a COVID-19 immunization campaign). Therefore, DBS collection and anti-IgG serology show promise as a tool to measure SARS-CoV-2 seropositivity at the population level. The benefits include comparable diagnostic accuracy to that of serum samples, with improved population reach and the potential to discriminate natural from vaccine-elicited seroconversion by measuring anti-S and anti-N IgG reactivity in a single reaction (20). In a theoretical population with 75% COVID-19 seroprevalence, a positive DBS-MSD result reliably indicates seropositivity with a high PPV and does not require additional testing. The lower NPV indicates that a negative result does not reliably predict lack of an immune response or immunosenescence. We suggest reflex testing a negative DBS result in a high seroprevalence setting when confirmation of an individual’s serostatus is required (21). Reflex testing could occur using venipuncture or an additional DBS. Applying predictive values requires Bayesian inference because seroprevalence changes over time (22). For example, serological testing too soon or long after antigen exposure will not accurately predict seroconversion.

An important limitation of SARS-CoV-2 seroprevalence studies in populations with low vaccine coverage is the difference in antibody signals between individuals who have recovered from asymptomatic and symptomatic infection (23). The distributions of serological signals between recovered asymptomatic and negative cases are more likely to overlap than when comparing negative to recovered symptomatic nucleic acid amplification test-confirmed cases. Low serological signal should not be a limitation in populations with high vaccine coverage, as SARS-CoV-2 vaccination has been experimentally found to elicit a stronger humoral immune response than natural infection (24). Our work corroborates the positive relationship between vaccination and anti-S IgG concentration because the sensitivity of the MSD assay increased when used in COVID-19 vaccine dose one and two recipients (Fig. 3).

In summary, measuring SARS-CoV-2 seropositivity at the population level presents unique challenges, which warrant investigation and consideration of alternative methodologies. We show robust diagnostic accuracy of DBS samples when tested for anti-SARS-CoV-2 IgG using an MSD assay and model the predictive value of DBS-MSD testing in a theoretical population with 15, 45, and 75% seroprevalence (e.g., COVID-19 vaccine coverage) (25). DBS tests have comparable sensitivity and specificity to those conducted on serum, regardless of low analyte volume. The PPV of DBS-MSD testing increases in response to high seroprevalence, making it possible to accurately identify individuals who have a humoral immune response. The NPV decreases as the expected prevalence increases, necessitating reflex testing for confirmation of true negatives. Seroprevalence differs over time due to time-varying antigen exposure in the community and antibody waning (24). As such, the predictive value of the assay is also liable to change as a function of symptom onset or vaccine administration to collection time (22). We observed the sensitivity of DBS-MSD testing to increase in an immunized population. This agrees with the finding that vaccinated individuals who received an mRNA-1273 vaccine (SPIKEVAX) possess a more robust humoral response than those that are naturally infected (24). The effect of vaccination on the humoral immune response may differ by vaccine type, number of doses, regimen, and other host factors (26).

At the population level, naturally infected and vaccinated individuals can be considered a homogenized group, where an anti-S IgG-positive signal indicates seroreactivity. Conversely, individual diagnosis may require further interpretation where anti-S IgG-positive persons are further stratified by their anti-N IgG results and/or clinical information (e.g., vaccination status and prior laboratory results) to determine natural infection from vaccine-elicited immunity (27). Detection of natural infection by serology alone depends on study design because anti-N IgG signal wanes when the time between exposure and sample collection lengthens (28, 29).

Public health practitioners should consider the utility of DBS testing by MSD to evaluate SARS-CoV-2 seropositivity from natural infection or COVID-19 vaccination (30). Modeling shows that this combination possesses a strong PPV (∼98%) in settings of high seroprevalence (e.g., 75% COVID-19 vaccine coverage). Public health agencies are challenged with simultaneously administering COVID-19 vaccines and measuring the elicited immune response. Addressing the latter requires a reliable and accessible method for detecting SARS-CoV-2 seropositivity (17). The logistic, economic, and demonstrated diagnostic accuracy of DBS-MSD testing make it a strong candidate for population-level investigation of SARS-CoV-2 antibody responses, especially in longitudinal study designs requiring repeated laboratory measures.

## MATERIALS AND METHODS

### Participant sampling.

This cross-sectional study includes samples from several COVID-19 seroprevalence studies conducted in British Columbia during 2020 and 2021 (Table 2). Samples from across these studies were merged into an analytic data set to conduct analysis (*n* = 6,841). The sample base and exclusion criteria differ between studies, and resampling between them increases the robustness, validity, and veracity of our estimates. All the laboratory specimens (serum or DBS) were collected using a standardized protocol and centrally tested at the British Columbia Centre for Disease Control Public Health Laboratory. Study descriptions are available in Table 2.

**TABLE 2:** Descriptions of cross-sectional serological surveys conducted during 2020 or 2021 in British Columbia from which nonhospitalized participants were sampled to construct an analytic data set (*n* = 6,841)

Study name^*a*^	Primary investigator(s)	Sample base	Enrollment period	Inclusion^*b*^ and exclusion^*c*^ criteria	No. of participants sampled
ASSESS-DBS	Muhammad Morshed	Incarcerated persons or workers in British Columbia Provincial Correctional Centers	January 2021–February 2021	One or more dose COVID-19 vaccine recipients^*c*^	*n* = 619
PREVENT-COVID	Agatha Jassem and Manish Sadarangani	Adults residing in British Columbia who are due to receive a COVID-19 vaccine	February 2021–ongoing	≤18 yrs of age^*b*^	*n* = 90
SPRING	Manish Sadarangani	Children and young adults residing in British Columbia	September 2020–ongoing	≥25 yrs of age^*b*^	*n* = 2,036
RESPPONSE	Lori Brotto and Gina Oglivie	Adults residing in British Columbia	November 2020–July 2021	<25 or >69 yrs of age^*b*^	*n* = 4,073
Biobank samples	David Goldfarb	Residents of British Columbia	November 2020–April 2021	PCR test negative for SARS-CoV-2 infection^*c*^	*n* = 23
Analytic data					*n* = 6,841

a Demographic variables like age or biological sex were not provided by study administrators and, therefore, were omitted from our analysis.

b Describes study inclusion criteria.

c Indicates study exclusion criteria.

The University of British Columbia Clinical Research Ethics Board provided ethical review and approval for studies from which participants were included (H20-02184, H20-02402, H20-01421, H20-03951, and H20-01886).

### Specimen preparation and storage.

Serum samples (*n* = 642) were collected from venipuncture by trained phlebotomists in 5-mL tubes (BD vacutainer SST tubes, 367986), centrifuged, and tested before storage at −20°C. DBS samples (*n* = 6,199 unpaired and *n* = 642 paired with serum) were collected by capillary finger prick using a contact-activated lancet (BD microtainer, 366594), spotted on protein saver cards (Whatman 903, Z761575), sealed in a gas-impermeable sachet with 1 gm of desiccant per card, and stored at −20°C. DBS sample collection was performed by a health care worker or by self-collection. Written instructions were provided to participants who were asked to self-collect. Four 6-mm punches were eluted in 350 μL of dipotassium phosphate-buffered saline with 0.5% sodium azide and 1.5% bovine serum albumin (wt/vol) (Ortho Clinical Diagnostics, personal communication).

### Serological testing.

Ten microliters of serum or DBS eluate was diluted 1:5,000 (vol/vol) or 1:500 (vol/vol) in Diluent 100 (MSD, R50AA-2) before testing (14, 31). Serological testing was performed with the V-PLEX COVID-19 coronavirus panel 2 (IgG) (MSD, K15369U), adhering to the manufacturer’s protocol (14). Reference positive samples were defined as paired serum samples with signals above the MSD-recommended target thresholds for anti-spike (S) and/or anti-nucleocapsid (N) and/or anti-receptor-binding domain (RBD) IgG (S = 1,960, N = 5,000, and RBD = 538) (14). Serum samples were classified as positive for SARS-CoV-2 anti-IgG when signal for two of three targets was greater than or equal to the threshold (32). Agreement between targets has better efficiency and increased sensitivity to detect low-level antibody responses than a single epitope (33). Thresholds were set for DBS-MSD results by plotting distributions of anti-S and anti-N IgG signals. DBS-MSD samples were classified as positive if the anti-S and anti-N signals or only the anti-S signal was greater than or equal to the thresholds established for DBS samples. Nucleocapsid-only positive DBS-MSD samples were classified as negative.

### Anti-SARS-CoV-2 IgG linear range.

The linear range of the MSD assay for DBS samples was determined by plotting the anti-IgG target signal stratified by viral antigen (S, N, or RBD) against the antibody concentration in arbitrary units per milliliter (AU/mL), which may be converted to international standard units per viral antigen (18). Linearity of the assay’s performance was examined by linear regression (34).

### Threshold determination and diagnostic accuracy.

Signal thresholds for interpreting a DBS result were manually determined from the anti-IgG signal distributions. These thresholds were used to calculate the sensitivity and specificity of a DBS-MSD test result in comparison to the paired serum reference (*n* = 642) by logistic regression (35).

### Discrimination.

Discrimination of the logistic regression model was assessed by an *n* = 2,000 bootstrap of the paired data (*n* = 642) and plotted as a receiver operator characteristic curve (ROC) (36, 37).

### Cross-validation.

The DBS-MSD anti-S threshold was cross-validated in a sample of prevaccinated and vaccinated participants taken from the PREVENT-COVID study (*n* = 90) (Table 2). This sample was not included in the data set from which the thresholds were determined; therefore, the logistic regression model was trained in a sample of unvaccinated participants and tested in vaccinated ones (38). The anti-N threshold was excluded from the cross-validation, as COVID-19 vaccination does not elicit anti-N humoral immunity (27). A two-way analysis of variance (ANOVA) was used to investigate the relationship between vaccination status and mean anti-S IgG concentration in log_10_ AU/mL.

### Prevalence and predictive value.

Sensitivity and specificity estimates from the sample of unvaccinated and/or naturally infected participants (*n* = 642) or vaccinated ones (*n* = 90) were used to calculate the positive and negative predictive values (PPV and NPV, respectively) of DBS-MSD testing in a theoretical population (*n* = 10,000) with various seroprevalence of 15, 45, and 75% (25, 39) (Table S1 in the supplemental material). Additionally, the sensitivity and specificity estimates were pooled for a robust estimate of the DBS-MSD test’s predictive value (Table 1).

### Data analysis.

All data analysis was performed in R version 4.10 using the following packages: tidyverse, reshape, car, dataexplorer, pROC, publish, caret, jtools, and generalhoslem (40).
