# Supplementary material for: Performance of Immunoglobulin G Serology on Finger Prick Capillary Dried Blood Spot Samples to Detect a SARS-CoV-2 Antibody Response
Source: Microbiol Spectr. 2022 Mar 10;10(2):e01405-21. doi: 10.1128/spectrum.01405-21 (PMC9045222; doi:10.1128/spectrum.01405-21)
Supplement: SUPPLEMENTAL FILE 1 — Supplemental material. Download SPECTRUM01405-21_Supp_1_seq4.pdf, PDF file, 0.1 MB [file spectrum01405-21_supp_1_seq4.pdf]

Supplementary Data:

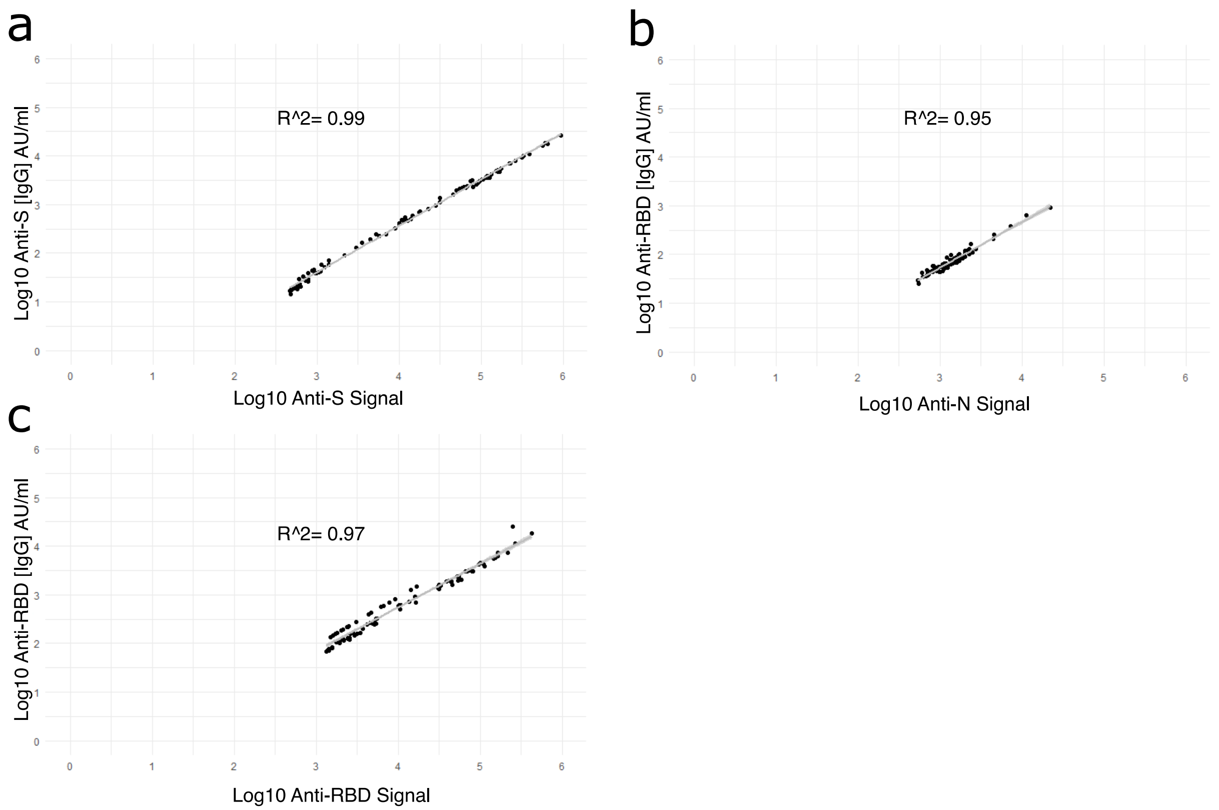

Figure S1: Linearity of the MSD V-PLEX COVID-19 Coronavirus Panel 2 (IgG) assay was examined in n = 90 samples from the PREVENT-COVID study (Table 1). Samples were collected from participants pre-vaccination (n = 30) or three to six weeks post dose one (n = 30), or two (n = 30) of a COVID-19 vaccine. Linearity was assessed for each antigen separately using linear regression, where the anti-body concentration (Log10 [IgG] AU/ml) is regressed on the diagnostic signal (light units). **a**) Anti-S IgG displays linearity and homoscedasticity across a wide dynamic signal range (~4 Log10 change) (R^2 = 0.99, P<0.001). **b**) Anti-N IgG displays linearity and homoscedasticity across a slightly smaller dynamic signal range (~3 Log10 change) (R^2 = 0.95, P<0.001), variance increases at greater signal values because natural infection is rare. **c**) Anti-RBD displays co-linearity with Anti-S IgG, with slightly more variance across a ~3 Log10 range (R^2 = 0.97, P<0.001).

Sample (Study)	Estimate		Prevalence (Vaccine Coverage %)		
	Sensitivity (95%CI)	Specificity (95%CI)	*15%* Estimate (95% CI)	*45%* Estimate (95% CI)	*75%* Estimate (95% CI)
**Un-Vaccinated (ASSESS) n= 624**	79% (58-91%)	97% (95-98%)	82% (80-84%)	96% (95-96%)	99% (98-99%)
**Pre-Vaccination (PREVENT) n = 30**	--	90% (73-98%)	--	--	--
**Dose One (PREVENT) n=30**	97% (83-99%)	--	63% (61-65%)	88% (87-90%)	96% (96-97%)
**Dose Two (PREVENT) n=30**	100% (88-100%)	--	64% (62-66%)	89% (88-90%)	95% (94-95%)

Table S1: Individual estimates of positive predictive value from unvaccinated, pre-vaccinated, dose one and dose two COVID-19 vaccine recipients. Sensitivity and specificity estimates are applied over a range of seroprevalences (fifteen, forty-five and seventy-five percent) in a theoretical population of ten-thousand people (n=10,000). The positive predictive value increases when the expected prevalence is high. The sensitivity of DBS-MSD testing to detect seroreactivity increases in vaccinated persons when compared to an un-vaccinated group with natural infection.

Sample (Study)	Estimate		Prevalence (Vaccine Coverage %)		
	Sensitivity (95%CI)	Specificity (95%CI)	*15%* Estimate (95% CI)	*45%* Estimate (95% CI)	*75%* Estimate (95% CI)
**Un-Vaccinated (ASSESS)**	79% (58-91%)	97% (95-98%)	96% (96-97%)	85% (84-86%)	61% (59-62%)
**Pre-Vaccination (PREVENT)**	--	90% (73-98%)	--	--	--
**Dose One (PREVENT)**	97% (83-99%)	--	99% (99-100%)	97% (97-98%)	75% (73-76%)
**Dose Two (PREVENT)**	100% (88-100%)	--	100% (99-100%)	100% (99-100%)	100% (99-100%)

Table S2: Individual estimates of negative predictive value from unvaccinated, pre-vaccinated, dose one and dose two COVID-19 vaccine recipients. Sensitivity and specificity estimates are applied over a range of seroprevalences (fifteen, forty-five and seventy-five percent) in a theoretical population of ten-thousand people (n=10,000). The negative predictive value increases when the expected prevalence is low.
